# Group B streptococcus late-onset disease,contaminated breast milk and mothers persistently GBS negative: report of 3cases

**DOI:** 10.1186/s12887-018-1192-x

**Published:** 2018-07-05

**Authors:** Giangiacomo Nicolini, Martina Borellini, Vitaliana Loizzo, Roberta Creti, Luigi Memo, Alberto Berardi

**Affiliations:** 1 Unità Operativa Complessa di Pediatria e Patologia Neonatale, Ospedale San Martino, Belluno, Italy; 2 Dipartimento Salute della Donna e del Bambino, Scuola di Specializzazione in Pediatria, Università di Padova, Padova, Italy; 3 Scuola di Specializzazione in Pediatria, Università degli Studi di Modena e Reggio Emilia, Modena, Italy; 4 Dipartimento di Malattie Infettive, Istituto Superiore di Sanità, Roma, Italy; 5 Unità Operativa di Terapia Intensiva Neonatale, Dipartimento Integrato Materno-Infantile, Azienda Ospedaliero-Universitaria Policlinico, Modena, Italy

**Keywords:** Breastfeeding, Group B *streptococcus*, Newborn, Sepsis

## Abstract

**Background:**

Human milk is fundamental for its nutritional properties and to protect newborns, but it is not sterile and can sometime transmit bacteria. Few anecdotal cases suggest that breast milk could be a possible source of group B *Streptococcus* (GBS) late onset disease, although the pathogenesis is not entirely understood.

**Case presentation:**

We report 3 cases of GBS late onset disease in full-term newborns. Fresh breast milk cultures yielded GBS, but mothers of neonates had no signs of mastitis and remained persistently GBS negative at rectovaginal site.

**Conclusions:**

Breast milk containing group B *Streptococcus* can be a risk factor for late onset disease. The persistent negative maternal GBS status supports the assumption that newborns, colonised in the throat, could be the initial source of GBS, while the mammary gland could act as a GBS replication site. It is unclear whether a low bacterial load may represent only contamination rather than true milk infection.

## Background

Breast milk has primary importance for feeding of the newborn, because of its nutritional properties and the contribution to the development of host defences [1]. However, human milk is not sterile and can sometime transmit bacteria. Group B *Streptococcus* (GBS) is a leading cause of neonatal infections in developed countries [2]. Two distinct syndromes are recognized: early-onset disease (EOD, from birth to day 6) and late-onset disease (LOD, from day 7 to 89) [3]. Several case reports have suggested breast milk as a possible source of GBS LOD [4]. Nevertheless, the mechanisms of GBS transmission and LOD pathogenesis are not yet clear. GBS gastrointestinal and genitourinary tract colonisation is common, and it ranges in pregnant women from 4 to 36% [5, 6]. However, only 0.8 to 3.5% of mothers carry GBS in their breast milk [7, 8].

We report 3 cases of LOD in full-term newborns who were breastfed with GBS contaminated milk. The newborns’ mothers had no signs of mastitis and their rectovaginal swabs were permanently GBS negative (both at prenatal screening and at the time of diagnosis of LOD). These findings suggest that the transmission may occur through a circular mechanism: the newborn (colonised in the throat) could be the initial source of GBS, while the mammary gland could act as a GBS replication site.

## Case presentation

### Case 1 (Table 1)

A Caucasian male neonate was born at 38 weeks’ gestations by vaginal delivery. Rectovaginal screening culture was negative for GBS. Birth weight was 3290 g; Apgar score was 9 and 10 at 1 and 5 min respectively. The newborn suffered from a mild tachypnoea on day 1, but was healthy and breastfed when discharged home on day 3. On day 9, he was admitted to the emergency department because of irritability and poor feeding. At admission, heart rate was 220 bpm. Blood testing showed mildly raised levels of lactate (3.9 mmol/l), and procalcitonin (PCT, 3.24 μg/L). Broad spectrum antibiotics (ampicillin and cefotaxime i.v.) were promptly given after cultures collection. Urine test, chest and abdominal ultrasounds found no source of infection. GBS was cultured in blood and in breast milk although the mother had no signs of mastitis. Maternal rectovaginal and urine cultures were sterile. Breastfeeding was not discontinued but milk culture became sterile after a 7 day course of oral amoxicillin was given to the mother. The newborn promptly improved and after 7 days i.v. antibiotics were shifted to oral amoxicillin that was discontinued on day 10. No recurrence of GBS was observed. Both GBS strains isolated from neonatal blood and milk were serotype III.

### Case 2

A male neonate was born at 37 weeks’ gestation after vaginal delivery to a Caucasian woman. Antenatal GBS screening was negative. Birth weight was 3375 g; Apgar score at 5 min was 10. The newborn was healthy and breastfed when discharged home. On day 17, he was admitted to the emergency department because of poor feeding, irritability and fever (*T* > 38 °C). Broad spectrum i.v. antibiotics (ampicillin and gentamicin) were given after cultures collection. Laboratory tests showed severely raised CRP levels (22 mg/dl) and CSF WBC (8000/mm^3^, with predominance of polymorphonuclear cells). Brain ultrasound study revealed a mild enlargement of lateral ventricles. Blood, CSF and fresh breast milk cultures yielded GBS. Maternal rectovaginal and urine cultures were sterile and the woman had no signs of mastitis. The infant was discharged home after a 14 days course of i.v. ampicillin and had no further relapses of LOD. Both neonatal and maternal GBS isolates were serotype III and had an identical genetic profile by Pulsed Field Gel electrophoresis [9].

### Case 3

A female was delivered vaginally (at 40 weeks’ gestation) to a Caucasian woman whose GBS screening culture was negative. The neonatal birth weight was 3176 g and Apgar score at 5 min was 10. She was healthy and breastfed when discharged home. On day 8 of life, the baby presented at the emergency department with fever, poor feeding and tachycardia. Laboratory tests showed raised CRP levels (13,5 mg/dl). Broad spectrum antibiotics (ampicillin and gentamicin) were given i.v. after collecting cultures (blood and CSF). Lumbar puncture showed low glucose levels (9 mg/dl), raised protein levels (300 mg/dl), and 6700 cells/mm^3^ with predominance of polymorphonuclear cells (95%). Blood culture was sterile whereas CSF and fresh breast milk cultures yielded GBS (80.000 CFU/ml in breast milk). The baby was given a 12 days course of ampicillin and was discharged home 14 days after admission. No recurrences were observed. The mother had no signs of mastitis and she was confirmed GBS-negative at rectovaginal site at the time of diagnosis of LOD.

**Table 1:** Demographics and clinical findings of the three cases of LOD and their own mothers

	CASE 1	CASE 2	CASE 3
Gestational age, *weeks*	38	37	40
Sex	M	M	F
Birth weight, *g*	3290	3375	3176
5 min Apgar score	10	10	10
Antenatal GBS screening	Negative	Negative	Negative
GBS rectovaginal culture at diagnosis	Negative	Negative	Negative
Breastfeeding	Yes	Yes	Yes
Days at presentation of late-onset disease	9	17	8
Symptoms	irritability, poor feeding	irritability, poor feeding, T° > 38 ° C	irritability, poor feeding, T° > 38 °C
Blood culture	GBS +	GBS +	GBS -
CSF culture	Not done	GBS +	GBS +
Day of antibiotics	10	NA	12
Fresh breast milk culture	GBS +	GBS +	GBS +
Mastitis	No	No	No
Antibiotics given to the mother	Yes	No	No
Breastfeeding during LOD	Yes	NA	NA
Fresh milk culture after antibiotic therapy	negative	NA	NA
GBS neonatal serotype	III	III	NA
GBS maternal serotype	III	III	NA
Lenght of stay, *days*	10	14	14
Recurrence	no	no	no

*GBS* Group B streptococcus, *F* Female, *LOD* Late-onset disease, *M* Male, *T°* Temperature, *NA* Not available

## Discussion and conclusions

This report deals with three cases of LOD in full-term neonates possibly attributed to the ingestion of breast milk containing GBS. Cases presented with sepsis and/or meningitis at day 9, 17 and 8, respectively. None of the mothers had signs of mastitis and all were GBS-negative at rectovaginal site (both at screening and at the time of diagnosis of LOD). In case 1 and case 2, neonatal and maternal GBS isolates were serotype III. Milk bacterial count was available only in case 3.

Berardi et al. evaluated GBS colonisation in 160 mother-baby pairs. GBS was identified in 53 neonatal throat cultures and 77 neonatal rectal cultures. GBS in breast milk was associated with heavy neonatal colonisation [10]. However, the mechanism of transmission of LOD through breastfeeding is poorly understood. The retrograde theory hypothesizes that GBS, present in the infant’s throat, colonises the mammary ducts during breast-feeding. GBS load increases in the milk, and in turn the infant is infected during breast-feeding (circular mechanism) [4]. Alternatively, some authors suggest that GBS might reach the mammary gland through the translocation of bacteria from maternal gut via lymphatics [11].

A recent review of the literature analysed cases of LOD in which the breast milk was tested positive for GBS [4]. The review pointed out that the role of breast milk in LOD remains controversial, although the milk would be a more convincing source when LOD occurs in a neonate born to a GBS negative mother, delivered after planned CS and when nosocomial sources are not identifiable. The review also reported that less than half mothers with GBS in breast milk had mastitis [4] and that most mothers (59%) were GBS negative at antenatal screening. However, Berardi et al. showed that only a few mothers (~ 25%) of neonates with LOD were confirmed GBS negative at rectovaginal site when they were retested at the time of diagnosis of LOD [12]. Therefore, the proportion of mothers who actually carry GBS at rectovaginal site is certainly underestimated if mothers GBS negative at screening are not retested at the time of diagnosis of LOD.

In the current study, 3 cases of LOD occurred in 3 different hospitals, therefore GBS strains are certainly unrelated. All mothers were confirmed GBS-negative at rectovaginal site. Newborns could have been colonised with GBS at mucosal surface (throat) after birth (from caregivers or environmental sources) and subsequently they could have transmitted GBS to mother’s mammary gland. Indeed, in case 2, the GBS yielded from breast milk and neonate showed an identical genetic profile. In these cases a circular mechanism seems particularly suggestive, whereas a bacterial translocation from maternal gut is unlikely because of persistent negative GBS rectovaginal culture.

Transition from silent breast duct colonisation to active GBS multiplication depends on many factors, such as milk stasis and bacterial load. Some investigators found that mothers with mastitis had higher GBS bacterial load (1.000.000 CFU/ml), than mothers without mastitis (≤100.000 CFU/ml) [12]. The lower bacterial count could suggest contamination during sampling rather than bacterial active multiplication. In the current study, none of the mothers had evidence of mastitis and milk bacterial count, available only in 1 out of the 3 cultures, was 80.000 CFU/ml. Some studies reported a total bacterial count < 10^6^ CFU/ml as the physiological threshold of bacterial load in human milk [13, 14]. The origin of milk bacteria is still not well understood, but several studies confirmed the existence of a dynamic network between breast-milk and newborn’s oral microbiota [15]. The causative role of breast milk in LOD results from the interaction of several maternal and neonatal factors. Prematurity, immature immune system, bacterial load and lesions of the intestinal mucosal barrier are recognised risk factors for progression to infection after ingestion of breast milk contaminated with GBS [4, 16]. This study is subjected to some limitations. Although mothers were apparently negative, we can not firmly rule out that they had a light colonisation, undetected by rectovaginal cultures. However, this eventuality seems unlikely. Furthermore, we have no data regarding the genetic profiles of most GBS isolated from mothers.. Finally, surface cultures were not collected from family members and neonates. This additional information could contribute to understand modes and mechanisms of GBS transmission.

Identifying the underlying mechanisms of postnatal transmission of GBS will be crucial to prevent cases of LOD. To date studies have not recognized the predominant mode (maternal, nosocomial or community) of postnatal GBS transmission. GBS in breast milk can be a risk factor for GBS LOD. The persistent culture-negative maternal GBS status suggests the transmission through a circular mechanism, with the newborn (colonised at the throat) as the initial source of GBS, and the mammary gland as a GBS replication site. High or low bacterial load in breast milk might help distinguish cases in which breast milk is actually infected from cases where breast milk is only “contaminated” during sampling.

Testing maternal GBS rectovaginal status and collecting breast milk culture at the time of LOD diagnosis could help to shed light on the mechanisms of GBS LOD.

## Abbreviations

CFU: Colony-forming unit
CRP: C reactive protein
CS: Caesarean section
CSF: Cerebral spinal fluid
EOD: Early onset disease
GBS: Group B *Streptococcus*
LOD: Late onset disease
PCT: Procalcitonin
WBC: White blood cells
